# Patient perceptions of severe COPD and transitions towards death: a qualitative study identifying milestones and developing key opportunities

**DOI:** 10.1038/npjpcrm.2015.43

**Published:** 2015-07-09

**Authors:** Amanda Landers, Rachel Wiseman, Suzanne Pitama, Lutz Beckert

**Affiliations:** 1 Nurse Maude Hospice Palliative Care Service, Christchurch, New Zealand; 2 University of Otago, Christchurch, New Zealand; 3 Canterbury Respiratory Services, Canterbury District Health Board, Christchurch, New Zealand

## Abstract

**Background:**

Chronic obstructive pulmonary disease (COPD) is a slowly disabling illness, with functional limitations and a high burden of symptoms. Palliative care services focus on quality of life for those facing life-limiting illness. Patients with COPD often see their illness as a ‘way of life’, not as a life-threatening illness, which makes the interface difficult.

**Aims:**

The aim of this study was to explore the experience of patients with advanced COPD after a life-threatening event, particularly focusing on end-of-life issues.

**Methods:**

Qualitative methods were used to capture patient experiences. Patients admitted for noninvasive ventilation for COPD were recruited and interviewed in their homes following discharge. The interview schedule explored the participants' understanding of their illness, concerns and plans, exploring end-of-life issues and perceptions of palliative care.

**Results:**

Participants were recruited until themes were saturated. Six transition points or milestones emerged: loss of recreation, home environment, episodes of acute care, long-term oxygen treatment, panic attacks, and assistance with self-care were common themes throughout the narratives.

**Conclusions:**

Milestones accumulate in no particular order. They can be easily recognised and allow health professionals to develop a common language with their patients. In advancing COPD, milestones may trigger the reassessment of goals of care and integration of a palliative approach.

## Introduction

Chronic obstructive pulmonary disease (COPD) is a slowly progressive respiratory illness. It is characterised by the insidious onset of shortness of breath, pain,^1^ limitation of function^2^ and is punctuated by exacerbations. It is predicted that by 2020 COPD will be the third leading cause of death and the sixth leading cause of disability worldwide.^3^ Primary and secondary services deliver different approaches to these patients who are often repeatedly presenting to the acute setting.^4^

Palliative care has traditionally functioned within the oncology/cancer paradigm focussing on quality of life in advanced disease.^5^ Over the past decade, comparisons have been made between COPD and lung cancer with regard to symptom burden, education and access to community support.^1,2^ A lack of specialist palliative care provision for those suffering from nonmalignant conditions has been identified.^1,2,6–9^ Studies have emphasised the unmet needs of patients with severe COPD.^10^ However, the traditional cancer model of specialist palliative care does not meet these patients’ needs.^11^ It may be more appropriate to explore ways of delivering the palliative approach in a way that is meaningful to each individual.

Pinnock *et al*.^12^ began exploring this knowledge gap by reporting patients’ experience of living with COPD. Patients described COPD as a ‘way of life’; many see themselves as living with, not dying of, COPD. Patients and families adapt to the subtle worsening of symptoms over months and years. The patients’ story of COPD has no beginning, no entry point and, therefore, the thought of an exit point has no context. According to Pinnock *et al.*, it is a chaotic history, punctuated by sickness, then often improvement. It is unclear how people with end-stage COPD prepare for end of life against the background of an unpredictable illness trajectory.^9,13–15^ It has not yet been defined how a health system offers a palliative approach to these patients in an appropriate, timely and sustainable way.

This study was designed to explore patients’ perceptions of advanced COPD and their understanding of a palliative approach to care. Patients were interviewed after a life-threatening hospital admission requiring noninvasive ventilation (NIV). Patient narratives were used to identify potential transition points indicating patient understanding of advancing illness. Particular emphasis was placed on exploring end-of-life issues, including the language used by patients.

## Materials and methods

The Canterbury District Health Board, the second largest in New Zealand, services a population of about 500,000. It has developed an innovative service delivery model that has improved relationships between primary and secondary care by developing shared pathways for management of patients with common conditions.^16^

This qualitative study used grounded theory as its methodology to explore the research question because of its ability to explore subjective experiences, and it incorporates specific stories that illustrate complex interactions and cause/effect outcomes. Initially, inductive analysis was used to support the development of codes and categories that emerged from the content data, and then used a deductive approach to further order the data.^17^ Grounded theory is also concerned with constructing theory, as opposed to testing theory.^18^ This approach positions the research participants as the source of knowledge and places them as experts on what is being studied. The research questions are then drawn from the participants’ subjective experiences and hypotheses are generated from these data.

### Participant selection criteria

Patients with a diagnosis of COPD admitted to respiratory specialist services at Canterbury District Health Board for NIV were approached. Criteria for provision of NIV are based on international guidelines,^19^ for patients with an acute respiratory acidosis because of an exacerbation of COPD. Retrospective audits show that approximately 50% of patients die within 2 years and only 26% are alive at 5 years.^20^ The requirement for NIV thus serves as a marker of advanced disease and increased risk of death.

All the participants had lung function tests confirming COPD. Exclusion criteria for participation included active treatment for lung cancer, NIV requirement for an alternative diagnosis, non-English speaking and/or cognitive impairment.

Ethical approval was obtained by both the Nurse Maude Ethical Advisory Group and Southern Health and Disability Ethics Committee (URB/12/02/012/A05).

### Recruitment of participants

A senior respiratory registrar identified patients who had required NIV during their admission for an exacerbation of COPD. Patients were approached 1–2 days before the planned discharge, once they were in a stable physical state. A verbal explanation and information sheet were given. No patient declined an information sheet. Patients were informed that a research nurse would contact them 2 weeks from discharge to further discuss the study, and extend an invitation to participate. After consent, the research nurse interviewed patients in their homes. The interviews were conducted by the same research nurse between April and August 2012, and all interviews were audiotaped and transcribed verbatim.

### Development of the interview schedule

A semi-structured interview schedule was developed after discussion among the research team. Nine questions were used exploring the following areas: patients' understanding of their COPD, future concerns about the impact of their illness, perceived future health needs, quality-of-life markers, expectations of future care plans, concerns about hospitalisation, current discussions with doctors about end-of-life care, and their perceptions of palliative care. After the first interview was conducted, an additional question about the experience of NIV was included.

### Data analysis

Three researchers (AL, LB and SP) observed, examined and interpreted the transcribed interviews to identify important patterns, themes and interrelationships.^17,21–24^ To ensure that the analysis was of high quality, each cycle of coding, definitions of codes and negotiated themes were reviewed by all the four researchers. Analyses were undertaken using the software QSR NVivo 10 (Doncaster, Victoria, Australia).^17,23,24^

To determine themes from the interviews, the researchers coded the data in two cycles. The first cycle used structural coding to order the data by using a question-based code that ‘acts as a labelling and indexing device, allowing researchers to quickly access data likely to be relevant to a particular analysis from the larger data set’.^17,25,26^ Next, descriptive analysis was undertaken leading to nine subcategories emerging from the data.

The second cycle of coding then drew on theoretical coding. The purpose of using theoretical coding was to ensure that the codes, subcategories and categories were further refined. This ensured that each code, subcategory and category was clearly linked to make comprehensible sense of the data.^17^ This resulted in a reduction to six themes emerging from the data.

## Results

A total of 15 participants were recruited. During the analysis process, it was identified that themes were saturated and therefore no further participants were sought. All participants had severe COPD and presented to acute services in respiratory failure. Demographics are summarised in Table 1. The majority of patients were living with family, had long-term oxygen therapy and poor lung function.

From the narratives, six themes emerged, which identified transition points and changes in care needs. These themes included loss of recreation, home environment, episodes of acute care, oxygen treatment, panic attacks and assistance with self-care. They appeared to accumulate over time in no particular order. These themes will now be extrapolated in the following section.

### 1. Loss of recreation

Respondents commented that participation diminished in recreational activities such as bowling, basketball, cooking, reading, knitting, sewing and gardening. Participants recognised when these activities became restricted or longer viable.

Participants were mournful that the reduced level of activity affected the depth of their relationships with others.

The loss of recreational activities, including spending time with family and/or friends, was identified by the participants as a milestone of advancing COPD.

### 2. Home environment

Many respondents hoped their care could be undertaken in their own homes. Participants wanted to be surrounded by family, feel in control and be close to familiar items, routines or pets. In some instances, they felt that dying at home would be more peaceful. Others wanted to only remain at home until they were ‘too sick’, and not become a burden to their family.

Any change to the home environment, moving to a new house or to a residential care facility was described within a context of decline of health. For some participants, this meant moving into an apartment with access to nursing support, for others it meant moving to a smaller property.

Change in the home environment was identified as a milestone within their COPD journey, and was because of the impact on their ability to retain independence and/or good health.

### 3. Episodes of acute care

Some participants identified the need for acute hospital care to manage symptoms as a milestone. These participants expressed confidence in the hospital to reduce their physical symptoms and related anxiety. Acute hospital care was often seen as a haven or place of security.

Participants who were admitted to hospital believed they could not have been managed at home. They recalled the need for admission to intensive care unit, requiring bilevel positive airway pressure (BiPAP) or resuscitation. Participants explained how such interventions were required to keep them alive; however, the negative prognostic implication of these admissions were not explored by participants.

Participants described different degrees of acceptance of acute care, yet all identified the need for increasing acute care as a milestone.

### 4. Long-term oxygen treatment

All participants noted requirement of long-term oxygen treatment as a transition point in the management of their care.

Many had preconceptions about the benefits of oxygen and its impacts on symptoms. Participants expressed their frustration about lack of improvement in energy level and mobility, as well as the practical burden of the equipment.

Long-term oxygen therapy was identified as a milestone that would result in more dependency.

### 5. Panic attacks

Several participants described panic attacks as part of their COPD journey. Many identified strategies that worked best for them, such as slowing breathing down or having someone to talk to for reassurance.

For some participants, cognitive strategies were insufficient and medications were required to manage the anxiety.

Panic attacks, not breathlessness, were identified as a milestone by participants. Participants did not always initially volunteer these attacks unless specifically asked; however, panic episodes were used by patients as a marker of advancing disease.

### 6. Assistance with self-care

Participants mentioned difficulty completing specific tasks, such as shopping, cleaning, washing and cooking. Support from friends and family enabled some participants to remain in their home. These participants articulated a feeling of burden on their support system.

For most participants, the ability to take care of their own personal hygiene was paramount in maintaining their dignity. This included the ability to independently toilet, shower/bath and dress. Participants expressed frustration and disappointment when they could not perform these tasks. Retaining this independence was seen as integral to their wellness highlighted by a desire to maintain dignity throughout their illness.

Participants articulated the inability to self-care as a milestone in their COPD journey.

## Discussion

### Main findings

Although the initial aim of the study was to explore patients’ perceptions of advanced COPD and the impact of a life-threatening event, six recurring transition points emerged using qualitative methods to articulate the patients’ voice. While exploring patients’ health needs, limitations and plans for the future, it became clear that they did not see themselves as dying, but living with their illness.

Six milestones or transition points of deteriorating illness were identified. Loss of the ability to participate in recreational activities was frequently mentioned. Discussions about planning for future place of care happened despite patients not recognising that they are close to death. Patients had mixed feelings about the need for acute care, but it was expected to be a part of their future needs. Long-term oxygen therapy featured strongly in the narratives. It is interesting that breathlessness was not mentioned as a trigger for concern about health status. However, when breathlessness induced panic, this was significant. Needing assistance with self-care signified the worrying possibility of lost dignity and of being a burden to others.

### Interpretation of findings in relation to previously published work

Pinnock *et al.* ^12^ established the concept of patients with severe COPD not viewing themselves as dying but living with an unpredictable chronic illness. This study builds on this further by identifying an accumulation of losses and events that occur before death in this population. Reinke *et al*.^27^ compared patients with COPD with those with lung cancer, particularly focusing on transition points of their illness. They found that in those with COPD the transitions often involved more than a ‘one-time’ event, echoing the findings of this study. The application of the milestones may allow identification of those patients who would benefit from an integrative palliative and respiratory care approach for patients with advanced COPD, as described recently by Higginson *et al*.^28^

Interviewing patients with severe COPD, Cawley *et al*.^29^ drew on the patient story to describe a series of important events as potential triggers for a holistic review of needs. These are events that signify a progression of the disease and interventions required to address the consequences of worsening COPD. Our study does overlap with some of these triggers or milestones and focuses on the patient perspective.

**Figure 1:**
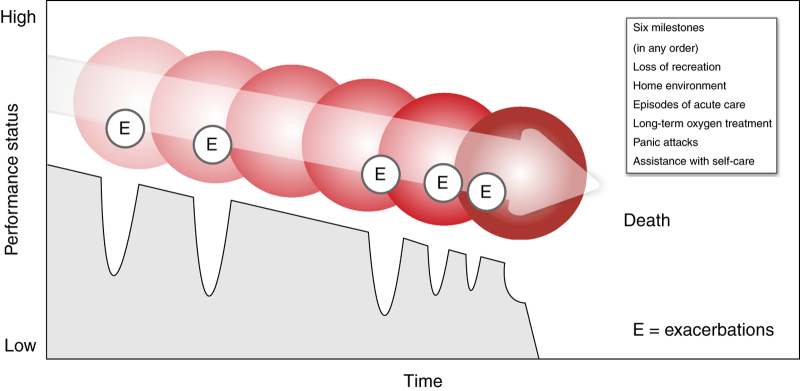
Trajectory of chronic obstructive pulmonary disease.

A new model of care is therefore proposed for patients with advanced COPD outlined in Figure 1. The accumulation of these milestones could be used to identify patients with severe COPD who are deteriorating. The patients may use the terminology outlined by the milestones when discussing their current health status with a health professional they trust. It highlights to the health care team a key opportunity to initiate vital end-of-life discussions and a transition to the palliative approach.

### Strengths and limitations of this study

These six milestones can be used in primary and secondary palliative care to target interventions such as symptom control for anxiety and breathlessness crisis plans at home. Significant improvements have recently been reported in breathlessness mastery from an integrated service offered by palliative care, allied health and respiratory medicine.^28^

Accumulating a number of milestones may indicate the need for a palliative approach and advance care planning. Specialist palliative care services would be involved in those with complex needs where the primary providers of palliative care require support, assessment and advice.

This study has several limitations. It was conducted to highlight the needs of patients with severe COPD and was not specifically designed to develop a new model of care. However, this model is based on patient interviews analysed using established qualitative methods to generate hypothesis.

### Implications for future research, policy and practice

This model of six milestones needs to be tested in different cohorts before it can be generalised to a wider population. Finally, we enrolled patients with advanced disease and life-threatening events; therefore, it is not certain whether this model will be applicable to patients with milder forms of COPD. Future research may involve questionnaire development and validation using the six transition points for use in identifying those patients with COPD and changing needs.

### Conclusions

Even patients with severe COPD see themselves as living with, and not dying of, COPD. Therefore, offering palliative and end-of-life support is difficult to initiate. However, patients described six milestones illustrating multiple losses that may serve as stimuli for patients and health services to change focus. Health professionals can use these key opportunities to plan together for the future.

## Figures and Tables

**Table 1:** Patient characteristics

*Demographics*	*N*=15
Sex, male/female	9/6
Age (mean, s.d., range)	69.2 (8.2, 55–89)
	
*Domiciliary status*	
Living alone	1
Living with family	10
Residential care	4
	
Long-term oxygen treatment	9
BMI (mean (s.d.))	24.7 (6.2)
	
*Severity of COPD*	
FEV_1_ (l; mean (s.d.))	0.69 (0.33)
FEV_1_% pred. (%; mean (s.d.))	26.4 (10)
PaO_2_ (mm Hg; mean (s.d.))	56.3 (10.9)
PaCO_2_ (mm Hg; mean (s.d.))	49.5 (9.2)
	
*Ventilatory status on admission*	
Arterial blood pH (mean (s.d.))	7.28 (0.06)
PaCO_2_ (mm Hg; mean (s.d.))	73.4 (0.06)
PaO_2_ (mm Hg; mean (s.d.))	53.9 (15.7)
